# Influence of Pneumococcal Vaccines and Respiratory Syncytial Virus on Alveolar Pneumonia, Israel

**DOI:** 10.3201/eid1907.121625

**Published:** 2013-07

**Authors:** Daniel M. Weinberger, Noga Givon-Lavi, Yonat Shemer-Avni, Jacob Bar-Ziv, Wladimir J. Alonso, David Greenberg, Ron Dagan

**Affiliations:** Yale School of Public Health, New Haven, Connecticut, USA (D.M. Weinberger);; National Institutes of Health, Bethesda, Maryland, USA (D.M. Weinberger, W.J. Alonso);; Soroka University Medical Center, Beer-Sheva, Israel (N. Givon-Lavi, Y. Shemer-Avni, D. Greenberg);; Ben-Gurion University of the Negev, Beer-Sheva (N. Givon-Lavi, Y. Shemer-Avni, D. Greenberg, R. Dagan);; Hadassah University Medical Center, Jerusalem, Israel (J. Bar-Ziv)

**Keywords:** pneumococcal conjugate vaccines, pneumonia, RSV, influenza, regression model, surveillance, viruses, Israel, respiratory syncytial virus, alveolar pneumonia

## Abstract

Postlicensure surveillance of pneumonia incidence can be used to estimate whether pneumococcal conjugate vaccines (PCVs) affect incidence. We used Poisson regression models that control for baseline seasonality to determine the impact of PCVs and the possible effects of variations in virus activity in Israel on these surveillance estimates. PCV was associated with significant declines in radiologically confirmed alveolar pneumonia (RCAP) among patients <6 months, 6–17 months, and 18–35 months of age (–31% [95% CI –51% to –15%], –41% [95% CI –52 to –32%], and –34% [95% CI –42% to –25%], respectively). Respiratory syncytial virus (RSV) activity was associated with strong increases in RCAP incidence, with up to 44% of cases attributable to RSV among infants <6 months of age and lower but significant impacts in older children. Seasonal variations, particularly in RSV activity, masked the impact of 7-valent PCVs, especially for young children in the first 2 years after vaccine introduction.

*Streptococcus pneumoniae* is a major cause of pneumonia worldwide, but in only a small fraction of severe cases are bacteria detectable in blood or cerebrospinal fluid (*1*). Because of the limitations of the diagnostic tools, identifying pneumococcal pneumonia is difficult and insensitive. Thus, using an endpoint of radiologically confirmed alveolar pneumonia (RCAP) can provide a more sensitive, but less specific, diagnosis of bacterial pneumonia that can be used to monitor the impact of pneumococcal conjugate vaccines (PCVs) on disease incidence (*2*,*3*).

The effect of vaccination on nonbacteremic pneumonia can be determined in the context of randomized control trials (*4*), in case–control studies (*5*,*6*), or by monitoring changes in the incidence of disease through routine surveillance (*7*). Of these options, surveillance data are the most readily available and give the most realistic estimate of vaccine effect, but they also are subject to biases, including secular trends and changes in detection or reporting (*8*,*9*). In the monitoring of nonbacteremic pneumonia incidence in children, a major source of variation might be year-to-year fluctuations in virus activity, which could influence the baseline and the post-PCV estimates of incidence (*7*,*10*).

Southern Israel provides a unique setting for evaluating the influence of PCVs on the incidence of pneumonia in children and the contributing role of respiratory viruses toward these estimates. The Jewish and Bedouin populations in this region inhabit the same geographic area but have limited social contact and generally differ in terms of socioeconomic status and illness patterns (*11*,*12*). Reflecting this diversity, there was little uptake of 7-valent PCV (PVC7) by the Bedouin population before the vaccine was introduced into the national immunization program in 2009 but moderate use among Jewish children on the private market. Since 2009, vaccine coverage has increased rapidly in both populations.

We sought to quantify the effect of PCV vaccination on RCAP incidence in southern Isreal. We took advantage of ongoing prospective studies of pediatric RCAP incidence and virus prevalence in children (*13*,*14*) that were conducted in the only hospital in southern Israel, which covers 95% of the population. We used Poisson regression models to estimate and control for the effects of seasonality and respiratory syncytial virus (RSV) and influenza activity on RCAP incidence and to determine the decline in incidence associated with increased uptake of PCVs.

## Methods

### Data Sources

RCAP cases were diagnosed at the Soroka University Medical Center, the only medical center in southern Israel, as described (*13*,*14*). The total population of children <3 years of age in the region in 2009 was ≈45,000, of whom 23,439 were Jewish children and 21,596 were Bedouin children. A case was defined as chest radiographic evidence of alveolar pneumonia in a patient <3 years of age who was seen in the pediatric emergency department or admitted to the hospital and in whom RCAP had not been diagnosed within the previous 28 days. Chest radiographs were analyzed according to the World Health Organization Standardization of Interpretation of Chest Radiographs Working Group (*15*). All readings were collected daily and read by 2 pediatric infectious diseases specialists (D.G. and R.D.) and independently confirmed by a pediatric radiologist, as described (*13*,*14*). The cases were aggregated into weekly time series for July 2004–June 2012 and stratified by ethnicity (Jewish vs. Bedouin) and by age group (<6 months, 6–17 months, and 18–35 months). Population size for each stratum was estimated on the basis of the number of births among the Jewish and non-Jewish populations in each calendar year in Beer-Sheva (*16*).

Uptake of PCV7 and 13-valent PCV (PCV13) was determined from an ongoing hospital survey starting in July 2009, when Israel’s national immunization program began covering PCV7. We enrolled the first 4 Jewish children and the first 4 Bedouin children <5 years of age seen on each working day at the pediatric emergency department of the Soroka Medical Center whose parents consented. The children’s vaccinating centers provided data on birth date, vaccination date, and whether the vaccine was PCV7 or PCV13. More than 95% of vaccinating centers responded to the questionnaires. During July 2009–April 2012, a total of 2,555 Jewish children and 2,666 Bedouin children were enrolled. Before the survey, no data were available on vaccine uptake. Health maintenance organizations (HMOs) began covering PCV7 in summer 2008; uptake among Jewish children was moderate (up to 25%) but was lower among Bedouin children. For the period before mid-2008, we assumed no vaccine uptake in either ethnic group. To fill in the values from the third quarter of 2008 through the second quarter of 2009, we assumed that coverage increased linearly from 0 in 2008 to observed vaccine uptake in 2009. We calculated the uptake in each quarter of the year and assigned this coverage to all weeks in that period.

Starting in July 2004, nasopharyngeal wash specimens were obtained from children during working days for virus testing. The decision to obtain nasopharyngeal wash specimens was made by the treating physicians and driven by clinical judgment. Specimens were obtained mostly from children admitted to the hospital and were processed in the virology laboratory within 6 hours. All samples were tested for RSV; influenza viruses A and B; and parainfluenza viruses 1, 2, and 3 by using either PCR or direct immunofluorescence antibody testing and commercial monoclonal antibodies (Chemicon, Temecula, CA, USA) supplemented by tissue culture (*17*,*18*). Weekly RSV and influenza counts were divided by the total number of swabs tested in that July–June year to adjust for biases in testing. These calculations were performed separately for Jewish and Bedouin children.

As an alternate measure of RSV activity, we used the weekly incidence of bronchiolitis among Jewish or Bedouin children. For this purpose, we retrieved from the hospital computer database all hospital visits (inpatients and outpatients) of children <3 years old for whom the final diagnoses included “bronchiolitis.” Monthly climate data on temperature, humidity, precipitation, and wind speed were obtained from the Central Bureau of Statistics (*16*) for the Beer-Sheva West meteorologic station.

### Estimating Vaccine Impacts

**Figure 1:**
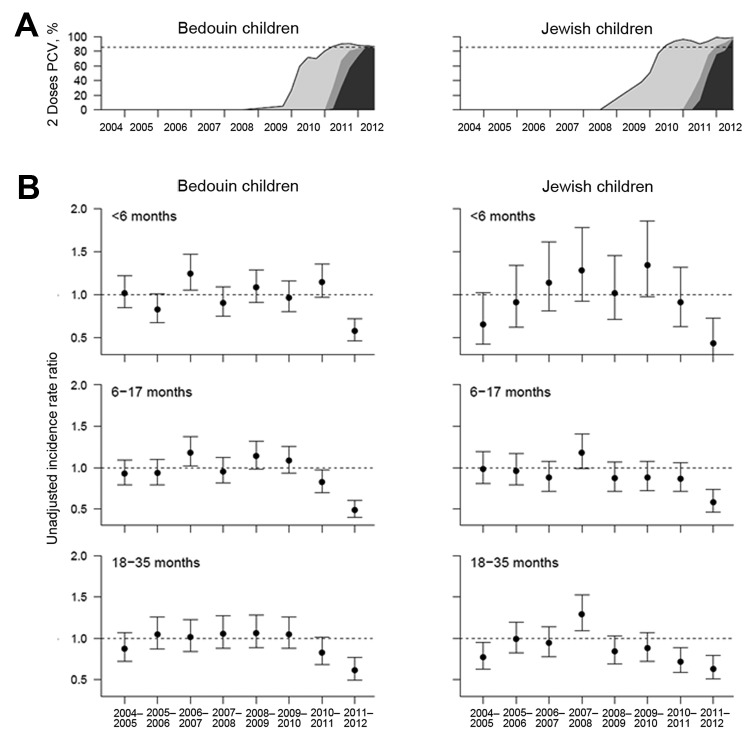
PCV uptake and decline in RCAP incidence 2004–05 through 2011–12, southern Israel. A) Uptake of >2 PCV doses among Bedouin and Jewish children 6–17 months of age. Black line indicates overall uptake; shaded areas show the proportion receiving >2 doses of PCV7 (light gray), >2 doses of PCV13 (dark gray), or >1 doses of PCV7 and 1 dose of PCV13 (medium gray). Dotted line indicates 85% uptake of any PCV. B) Unadjusted incidence rate ratio for RCAP comparing the incidence in each July–June year with the average for 2004–05 through 2007–08. RCAP, radiologically confirmed alveolar pneumonia; PCV, pneumococcal conjugate vaccine; PCV7, 7-valent PCV; PCV13, 13-valent PCV.

Unadjusted incidence rate ratios were calculated by determining the incidence in a given year (July–June) or given season (November–April or May–October) and dividing it by the mean incidence of the corresponding prevaccine period. To estimate the effects of RSV, influenza, and vaccine uptake on RCAP incidence, we fit a Poisson regression (log-link) model using PROC GENMOD in SAS version 9.2 (SAS Institute, Cary, NC, USA). The outcome variable was the weekly incidence of RCAP. The predictor variables were weekly activity of influenza and RSV among Jewish and Bedouin children (as described above), an indicator variable for ethnicity; sine and cosine terms that had frequencies of 52.25 and 26.125 weeks and varied between ethnic groups; and a binary variable that indicated whether the PCV vaccination program in the Jewish or Bedouin population had reached maturation. The vaccination program was considered to be mature when at least 85% of the Jewish or Bedouin populations 6–17 months of age had received at least 2 doses of any PCV (85% was chosen to indicate an immunization program with stable rates [Figure 1]). We also considered using vaccine uptake as a continuous variable in the model, but the relationship between vaccine uptake and incidence appeared to have a threshold effect, as might be expected given the role of herd immunity (Technical Appendix Figure 1).

This model assumes that the effects of the viruses and vaccination are the same for both ethnic groups but enables the seasonal baseline to vary in timing and intensity between ethnic groups. Periodic variables were selected on the basis of Akaike Information Criteria in preliminary fixed-effects analyses, and other variables were included if they were significant and improved the Akaike Information Criteria score. Interaction terms between ethnicity and the virus variables or vaccine uptake variable were tested but not included in the final model. The distribution of the deviance residuals was approximately normal for all strata except for the <6-month-old Jewish children (where the data were sparse).

We determined 95% CIs for the parameters using an 8-week moving-block bootstrap (n = 1,000) to account for autocorrelation in the time series (*19*,*20*). The incidence rate ratio associated with high vaccine uptake in each age group was estimated by taking the exponent of the regression coefficient for the vaccine uptake variable. The percentage change was estimated as 100 × (incidence rate ratio – 1); a negative percentage indicates a decline in incidence. The effects of influenza and RSV were calculated by fitting the model and then obtaining predicted values by substituting either the observed value for RSV or influenza or 0 into the equation. The difference between the estimates constituted the virus-attributed incidence, and the attributable percentage was the virus-attributed incidence divided by total estimated incidence. To validate the approach, we fit an alternative model in which the 2011–12 season was excluded or a model that excluded RSV and made predictions for the 2011–12 season. We also tested for variations in climate across the study period, such as monthly temperature and precipitation, that might be associated with the observed declines in RCAP incidence.

## Results

From 2004–05 through 2011–12, a total of 2,246 RCAP cases occurred among Jewish children <3 years of age and 3,690 cases occurred among Bedouin children <3 years of age (Table 1). Incidence was highest among the Bedouin children, and age at infection varied substantially between Jewish and Bedouin children (Table 1). For Jewish children, incidence peaked among 6–17-month-olds; for Bedouin children, among <6-month-olds. The incidence of RCAP (Table 1; Figure 1) and respiratory viruses (online Technical Appendix Figure 2) varied substantially between seasons.

**Table 1:** RCAP incidence, southern Israel, 2004–2012*

Ethnicity; age, mo.	RCAP, no. cases (incidence†)								
	Overall	2004–05	2005–06	2006–07	2007–08	2008–09	2009–10	2010–11	2011–12
Jewish									
<6	285 (9.1)	23 (6.2)	32 (8.7)	41 (10.8)	48 (12.4)	38 (9.6)	51 (12.8)	35 (8.6)	17 (4.1)
6–17	958 (15.6)	128 (16.9)	123 (16.6)	112 (15.2)	157 (20.7)	117 (15.1)	120 (15.2)	120 (15.0)	81 (10.1)
17–35	1,003 (11.0)	106 (9.6)	140 (12.3)	130 (11.7)	181 (16.3)	119 (10.5)	127 (10.9)	106 (9.0)	94 (7.9)
Bedouin									
<6	1,147 (39.9)	147 (41.9)	116 (33.9)	177 (51.1)	135 (37.9)	162 (44.5)	146 (39.6)	175 (47.0)	89 (23.7)
6–17	1,486 (26.0)	186 (25.7)	181 (25.8)	223 (32.6)	186 (26.9)	224 (31.4)	218 (29.9)	168 (22.8)	100 (13.5)
17–35	1,057 (12.4)	123 (11.5)	149 (13.7)	140 (13.3)	145 (14.1)	145 (14.0)	147 (13.8)	119 (10.9)	89 (8.1)

*RCAP, radiologically confirmed alveolar pneumonia. †Number of cases/1,000 children/year.

**Figure 2:**
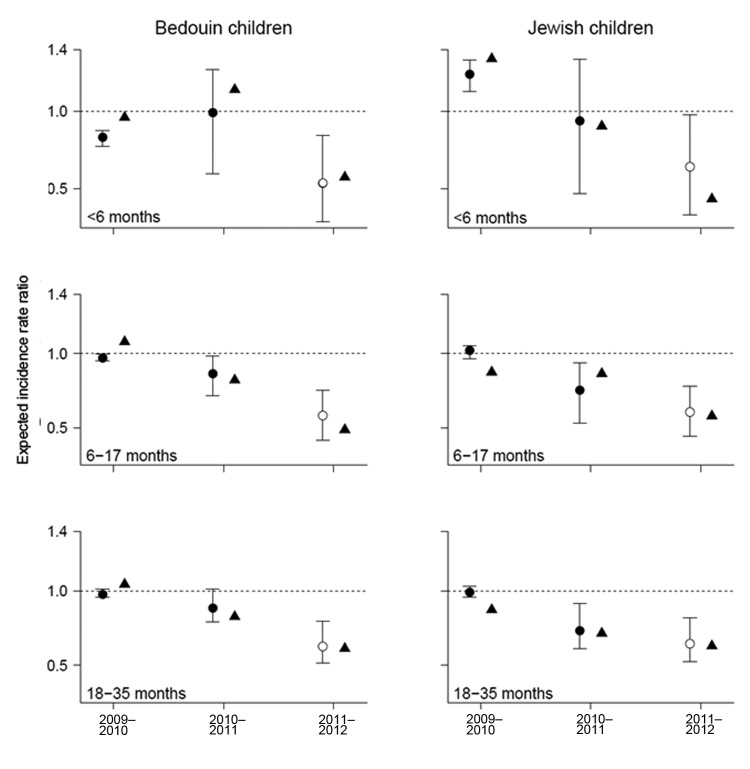
Observed (triangles) and expected (closed circles) change in RCAP incidence for each year compared with the 2004–05 through 2007–08 average, expressed as incidence rate ratios, southern Israel. Expected values were determined from a model fit to data from 2004–05 through 2010–11, with the 2011–12 values (open circles) extrapolated based on the observed virus activity.

### Changes in Alveolar Pneumonia Incidence, 2004–2012

We first evaluated whether the incidence of RCAP changed after PCV was introduced into the national immunization program. RCAP incidence decreased significantly in 2011–12 compared with the prevaccine era, by ≈49%, ≈46%, and ≈37% among Jewish and Bedouin children <6 months, 6–17 months, and 18–35 months of age, respectively (Figure 1, panel B). For the preceding year, 2010–11, RCAP incidence declined modestly among 18–35-month-old Jewish children (28%, 95% CI 11.0%–41%), Bedouin children overall (17%, 95% CI –1% to 32%), and 6–17-month-old Bedouin children (17%, 95% CI 2%–30%). However, RCAP incidence did not decline among Jewish children <6 months or 6–17 months of age, despite high uptake of the vaccine.

We also compared changes in incidence during winter months with changes in incidence during summer months, when there is little respiratory virus activity. RCAP incidence declined significantly in the summer of 2010 compared with the prevaccine era in the Jewish population but was similar to baseline levels during the following winter (Technical Appendix Figure 2, panel B). Among Bedouin children, where vaccine uptake was slower (Figure 1, panel A), RCAP incidence did not decline significantly until summer 2011.

### Association between RCAP Incidence and Activity of Respiratory Viruses

We next considered whether year-to-year variations in virus activity (Technical Appendix Figure 3) might influence estimates of vaccine impact and perhaps mask the effect of the vaccine during the first few years or lead to overestimation of vaccine effect during the weak RSV and influenza season of 2011–12. To evaluate the relationship between respiratory virus activity and RCAP incidence, we fit a regression model that accounts for virus activity, vaccine uptake, and baseline seasonal variations. RSV and influenza were associated with significant increases in RCAP incidence; RSV was associated with an especially strong increase. A total of 44% (95% CI 36%–52%), 21% (95% CI 15%–25%), and 16% (95% CI 11%–20%) of RCAP cases were attributed to RSV among children <6 months, 6–18 months, and 18–36 months of age, respectively (Technical Appendix Figure 4). Influenza was more modestly associated with RCAP incidence, with 1% (95% CI −6% to 6%), 4% (95% CI 1%–6%), and 2% (95% CI 1%–4%) of disease attributed to influenza among children <6 months, 6–18 months, and 18–36 months of age, respectively (Technical Appendix Figure 4).

When we controlled for virus activity, vaccination (>85% uptake) was associated with a significant decline in RCAP incidence of −31% (95% CI −51% to −15%), −41% (95% CI −52 to –32%), and −34% (95% CI −42% to −25%) among <6-month-old, 6–18-month-old, and 18–35-month-old children, respectively. Results from a model in which bronchiolitis hospitalizations, rather than RSV positivity, was used as a predictor gave similar results through the end of 2011 (data not shown). We also considered the possibility that the low RCAP seasons might be associated with anomalous climatic conditions but did not detect any significant differences in monthly temperature, humidity, precipitation, or wind across the study period that might explain the observed patterns; these variables were not included in the final model.

To evaluate the sensitivity of our results to the RCAP incidence in 2011–12, we refit the model to data from 2003–04 through 2010–11 and extrapolated the change in incidence for 2011–12. The model accurately predicted the observed change in RCAP incidence in 2011–12 for all age groups (Figure 2).

### Importance of Including RSV and Seasonal Variables in the Analyses

As a sensitivity analysis, we tested whether inclusion of RSV in the model influenced the estimates of vaccine impact. When fitting the model to the entire dataset (2004–05 through 2011–12), removing RSV from the model had little or no effect on estimates of vaccine impact in any of the age groups (Table 2). However, when we fit the model to data only from 2004–05 through 2010–11 (including the first 2 years of PCV7 use) and then evaluate the impact of excluding RSV from the model, we found that RSV substantially influenced the estimates in children <6 months old (Table 2). Removing RSV from the model modestly affected the estimate of vaccine impact among the 6–17-month-old children and had no detectable on the estimates among the 18–35-month-old children (Table 2). This finding suggests that RSV masked the impact of vaccination on RCAP incidence in young infants in the first full year after vaccine introduction.

**Table 2:** Impact of pneumococcal conjugate vaccine from different models, southern Israel*

Age group, mo.	Fit to 2004–05 through 2011–12				Fit to 2004–05 through 2010–11 (excluding 2011–12)		
	Full model	Model without RSV†	Unadjusted‡		Full model	Model without RSV†	Unadjusted‡
<6	−31.5 (−50.6 to −14.5)	−30.7 (−57.2 to 9.7)	−20.0 (−70.2 to 76.5)		−27.9 (−62.9 to 9.2)	−5.2 (−68.5 to 83.9)	+22.3 (−81.5 to +106.0)
6–17	−40.5 (−52.1 to −31.5)	−39.6 (−52.7 to −26.0)	−36.0 (−63.4 to 4.6)		−36.3 (−53.6 to −19.7)	−29.9 (−53.7 to –7.8)	−20.0 (−69.5 to +44.0)
17–35	−33.6 (−41.5 to −25)	−33.0 (−40.9 tp −23.1)	−31.3 (−47.8 to −8.4)		−33.3 (−45.0 to −16.0)	−29.1 (−44.2 to –10.8)	−25.4 (−55.0 to +11.1)

*Vaccine impact is the estimated percentage change in disease incidence associated with pneumococcal conjugate vaccine use. RSV, respiratory syncytial virus. †Model fit with all predictors described in the methods section except for RSV activity. ‡Model fit with predictors for vaccine uptake and ethnicity only.

We also fit a simple model that did not include variables for virus activity or seasonal (harmonic) terms. Again, when the model includes data through the 2011–12 season, the omission of these variables had little impact on the estimates of vaccine impact (Table 2). However, when the model was fit just to data through the 2010–11 season, the estimate of vaccine impact was closer to 0, especially among younger children.

## Discussion

We have demonstrated that introduction of PCVs into the national immunization program in Israel was associated with a significant decline in the incidence of RCAP among both Jewish and Bedouin children. RSV activity and RCAP incidence were strongly associated, and controlling for such variation, along with regular seasonal fluctuations, improved our estimates of vaccine impact in young children, especially in the immediate post–PCV implementation period. These analyses highlight a potential bias that can influence estimates of vaccine-associated declines, particularly with short surveillance follow-up periods.

Most alveolar pneumonia is believed to have a bacterial etiology, either alone or in combination with a virus (*14*,*21*–*23*). A recent study from this population in Israel demonstrated that 37% of children <1 year old and 14.7% of children 1–2 years old who had RCAP had detectable RSV (*2*). The results of our analysis also support a strong association between RSV activity and RCAP incidence, particularly in children <6 months of age. RSV could potentially increase the risk of developing secondary bacterial infections, but using these data, we were unable to determine whether the RSV-attributable RCAP cases resulted from RSV infections alone or from viral–bacterial co-infections.

A previous study in South Africa found that a 9-valent PCV was associated with a vaccine efficacy against radiologically confirmed pneumonia of ≈20% among HIV-uninfected persons (*24*). The higher impact of PCV estimated in our analyses could be because of the age distribution of case-patients and the more specific diagnosis of alveolar pneumonia, most of which is most likely bacterial. Additionally, the herd immunity effect will be stronger after licensure than in a randomized control trial (*25*). Vaccine effectiveness against pneumonia also will depend on which pathogens are prevalent in a given region and the etiologic fraction of each. Finally, our study population had high uptake of PCV13, which targets serotypes 1, 5, 7F, and 19A—types associated with RCAP in young children in our region (*13*).

The measured vaccine impacts presented here resulted both from direct and indirect (herd immunity) effects. The indirect benefit of pneumococcal vaccination on unvaccinated age groups has been well documented (*7*), and we did not try to disentangle the relative contributions of direct or indirect protection in our analyses.

Our results assume a relationship between RSV incidence and alveolar pneumonia that remained consistent across the entire study period. Use of PCVs can influence viral incidence resulting from bacterial–viral interactions (*26*), which possibly could bias our results. Although we did not detect such a trend in the RSV data in this population, if such a bias existed, then decreases associated with the vaccine might be attributed instead to the virus decline, and the estimated impact of the vaccine would be attenuated. The estimated effect of influenza was smaller than for RSV but might have been more pronounced if we had been able to estimate separate effects for seasons during which influenza A(H3N2) is severe and seasons in which influenza A(H1N1) and influenza B are comparatively mild. Examining the impact of PCVs on the incidence or severity of viral pneumonias would be a promising area for future research.

The measurement of vaccine uptake used here is based on a hospital survey, which could be biased if health care use differs by ethnicity or economic status. This hospital has the only emergency department in the region, and it serves all Jewish and Bedouin children in the region. The immunization survey also measured diphtheria–pertussis–tetanus vaccination and found >90% uptake among both Jewish and Bedouin children. Because vaccine uptake was included as a threshold variable in the model, the results are relatively insensitive to any potential biases in the uptake data.

We considered an alternative model with a continuous vaccine uptake variable. This model gave comparable estimates of vaccine impact in 2010 for the 18–35-month age group (−30% for continuous vs. −34% for binary). The estimated decline was smaller when we used the continuous vaccine uptake variable for children <6 months (−18% vs. −31%) and 6–17 months of age (−32 vs. −41%).

Focusing on RCAP as an outcome has advantages and disadvantages. The diagnosis of RCAP in this study follows a well-defined protocol (*14*) and has undergone quality control validations. However, focusing just on RCAP can underestimate the total impact of pneumococcal vaccination (*2*). Therefore, our estimates, although based on high-quality data, possibly underestimate the total impact of the vaccine.

This model does not explicitly estimate the contribution of pneumococcal disease to RCAP incidence. Rather, we assume that any changes in the postvaccine period are attributable to the vaccine or to variations in RSV or influenza activity. If additional factors not accounted for by the model caused a decline in RCAP, we would overestimate the impact of the vaccine. Likewise, we assume that the seasonal terms (harmonics) capture weekly variations not attributable to virus activity. If these seasonal terms do not fit the data well, they would overestimate the effects of the viruses.

Given our analytical methods, we cannot definitively say that RSV is an independent cause of alveolar pneumonia, which is typically considered to have a bacterial cause. Further work with case–control studies or other designs is needed to be done to establish a causal link. Likewise, because our study was an ecologic study, we cannot definitively attribute any decreases to the vaccine. Longer-term surveillance is needed to confirm that the rates remain lower than the historical baseline, taking into account virus activity and other possible confounding factors.

In the absence of bacteriologic data from these patients, fully determining the relative importance of PCV7 and PCV13 is impossible, especially given the short observation period before PCV13 was adopted. The observed incidence rate for each quarter suggests a substantial drop in RCAP incidence before PCV13 uptake was high (Technical Appendix Figure 1). On the basis of invasive pneumococcal disease (IPD) data from Israel, in children <5 years of age, PCV7 serotypes were reduced by 81% after PCV implementation, with an overall reduction in IPD incidence of 43% by the end of 2010 (*27*). The remaining PCV13 serotypes accounting for 72% of the residual disease (*27*). As a result, PCV13 introduction could account for a considerable additional drop in IPD incidence in 2010–11 and 2011–12 if RCAP followed a pattern similar to IPD. Furthermore, some of the additional serotypes in PCV13 have high case/carrier ratios and therefore might cause a large decrease in disease incidence (*13*).

We have shown that the incidence of RCAP significantly declined after PCV was introduced and that statistical models accounting for virus activity can help to improve interpretation of these data. Such analyses can be used in conjunctions with other methods, such as case–control studies, active population-based surveillance, and carriage studies, to evaluate the impact of vaccination.

Technical Appendix Pneumococcal conjugate vaccine (PCV) uptake and decline in radiologically confirmed alveolar pneumonia (RCAP) incidence by season; weekly RSV-positive samples among Bedouin and Jewish children; example of model fit and estimated contribution of influenza and respiratory syncytial virus to RCAP incidence among Bedouin and Jewish children; and relationship between PCV uptake among 6–17-month-old children and the unadjusted incidence rate ratio, southern Israel, 2004–05 through 2011–12.
