# Supplementary material for: Influence of Pneumococcal Vaccines and Respiratory Syncytial Virus on Alveolar Pneumonia, Israel
Source: Emerg Infect Dis. 2013 Jul;19(7):1084–91. doi: 10.3201/eid1907.121625 (PMC3713978; doi:10.3201/eid1907.121625)
Supplement: Technical Appendix — Pneumococcal conjugate vaccine (PCV) uptake and decline in radiologically confirmed alveolar pneumonia (RCAP) incidence by season; weekly RSV-positive samples among Bedouin and Jewish children; example of model fit and estimated contribution of influenza and respiratory syncytial virus to RCAP incidence among Bedouin and Jewish children; and relationship between PCV uptake among 6–17-month-old children and the unadjusted incidence rate ratio, southern Israel, 2004–05 through 2011–12. [file 12-1625-Techapp-s1.pdf]

Article DOI: http://dx.doi.org/10.3201/eid1907.121625

Influence of Pneumococcal Vaccines on Respiratory Syncytial Virus–associated Alveolar Pneumonia, Israel

Technical Appendix

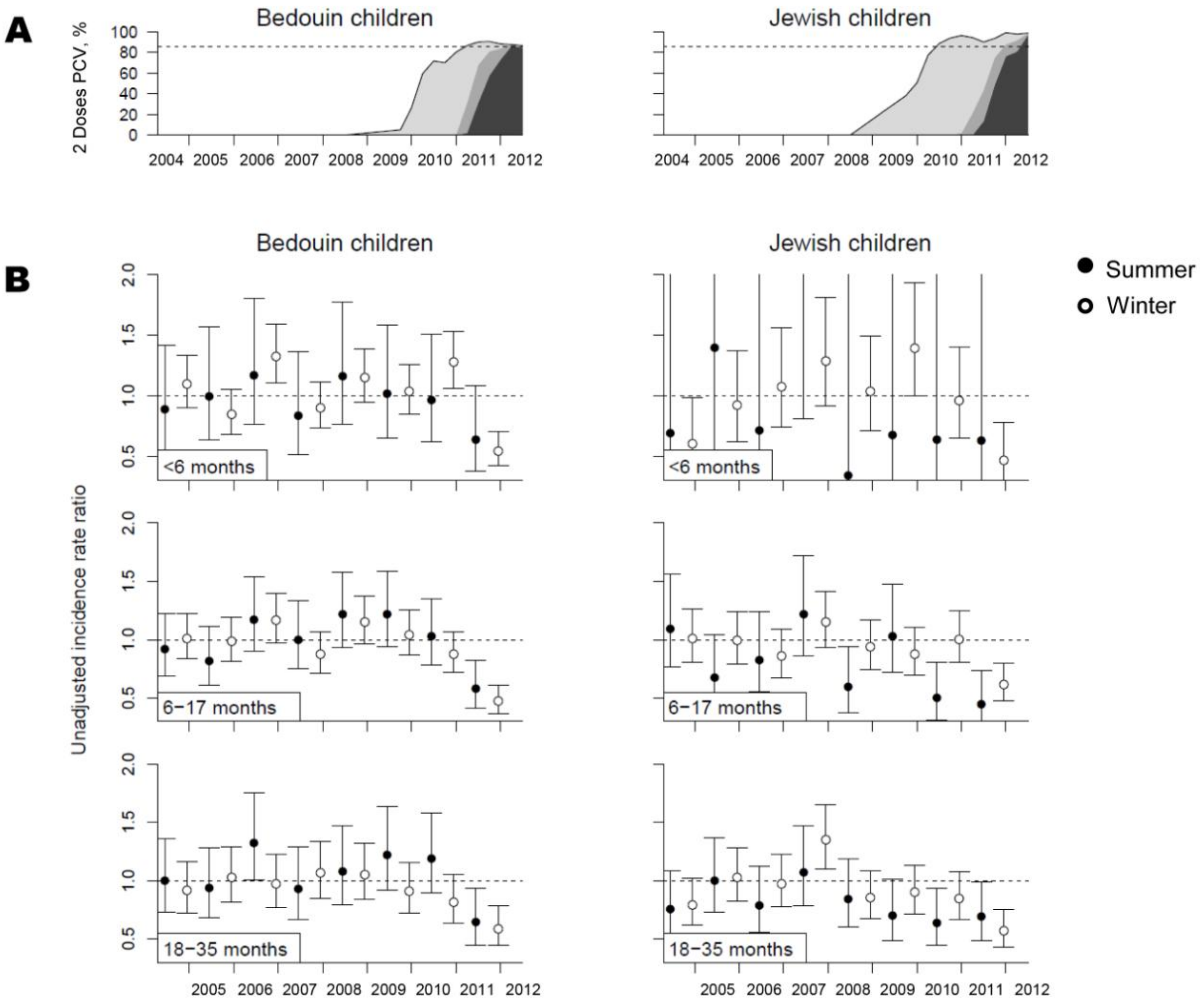

Technical Appendix Figure 1. Relationship between PCV uptake (≥2 doses of PCV7 or PCV13 among 6–17-month-old children) and the unadjusted incidence rate ratio (comparing the incidence in each summer or winter with the average incidence in the corresponding season for 2004–05 through 2007–08). The color of the marker indicates the percentage of vaccinated children (≥2 doses of PCV7 or PCV13) who

received at least 1 dose of PCV13. Squares, Jewish children; circles, Bedouin children. PCV, pneumococcal conjugate vaccine; PCV7, 7-valent PCV; PCV13, 13-valent PCV.

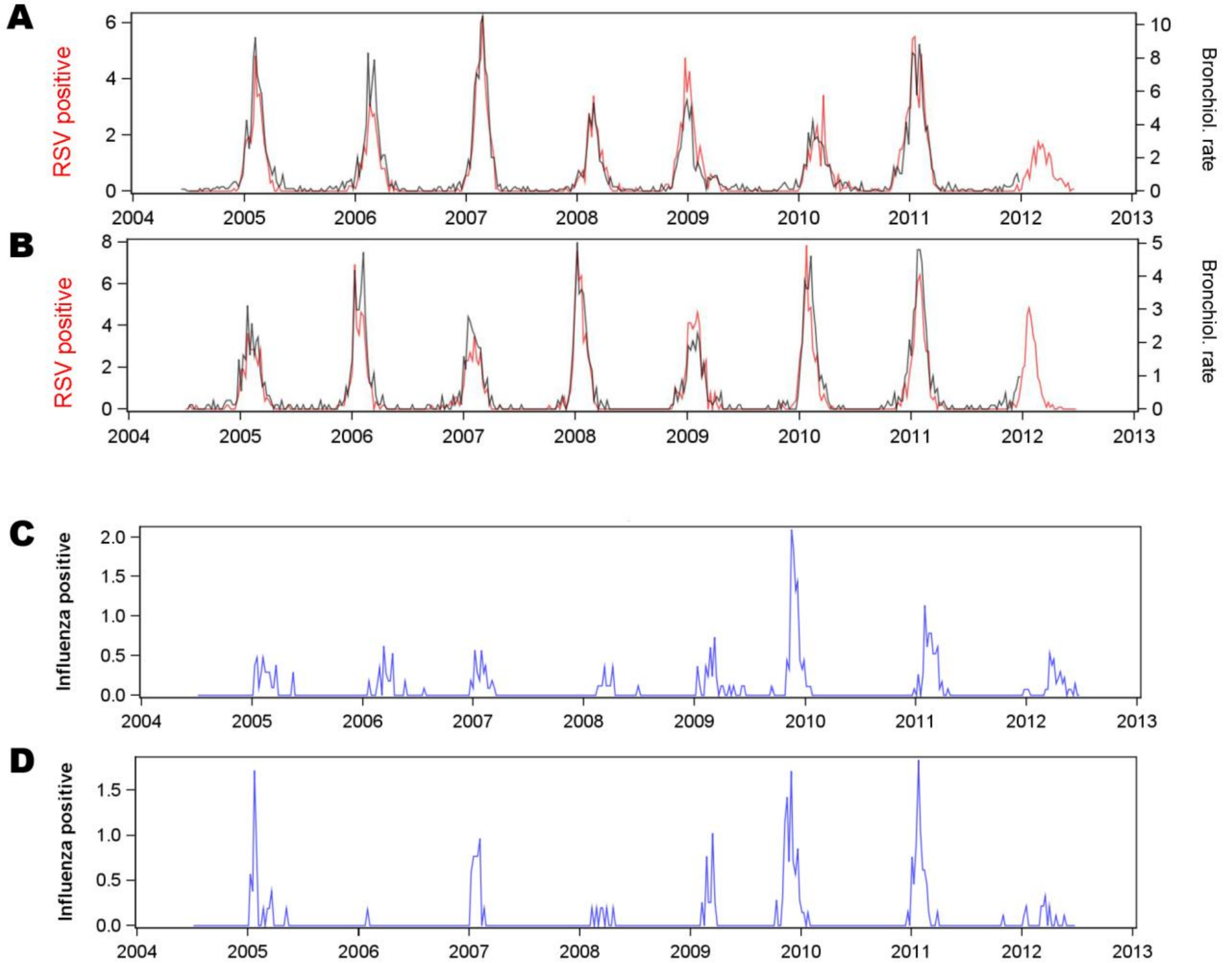

Technical Appendix Figure 2. PCV uptake and decline in RCAP incidence by (winter vs. summer, southern Israel, 2004–05 through 2011–12. PCV uptake and decline in RCAP incidence 2004 radiologically confirmed alveolar pneumonia 05-2011/12 by season (winter vs. summer). A) Uptake of ≥2 PCV dose among the Bedouin and Jewish children 6–17 months of age, southern Israel. Black line indicates the overall uptake; shaded areas show the proportion receiving ≥2 doses of PCV7 (light grey), ≥2 doses of PCV13 (dark grey), or ≥1 doses of PCV7 with 1 dose of PCV13 (medium grey). Dotted line indicates 85% uptake of any PCV. B) Unadjusted incidence rate ratio for RCAP by winter vs. summer, comparing the incidence in each winter (open circles) and summer (solid circles) with the corresponding average for 2004–05 through 2007–08. PCV, pneumococcal conjugate vaccine; RCAP, radiologically confirmed alveolar pneumonia; PCV7, 7-valent PCV; PCV13, 13-valent PCV.

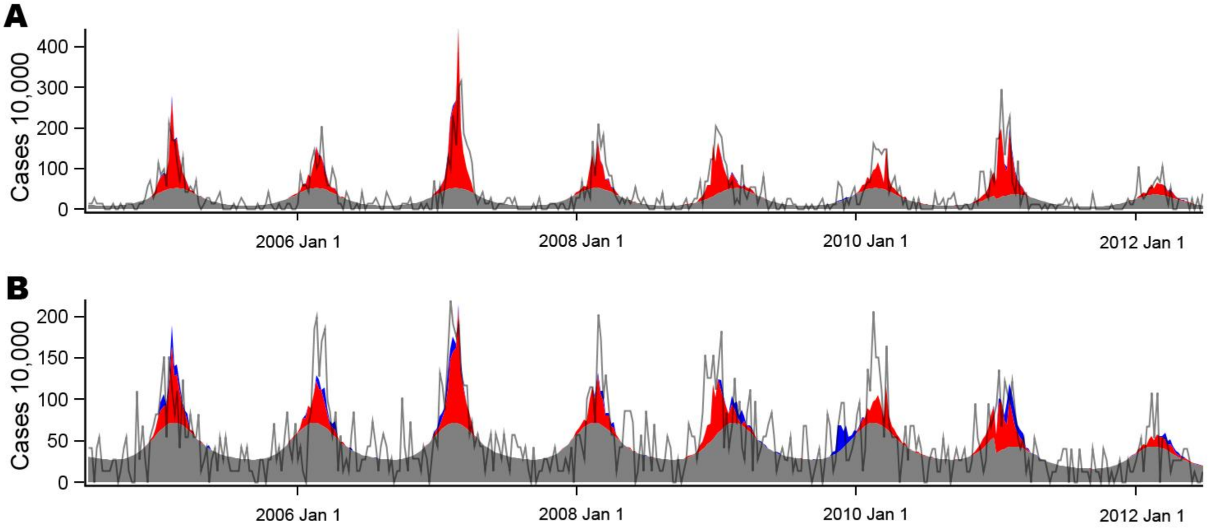

Technical Appendix Figure 3. Weekly RSV-positive samples (standardized to annual samples) (red) and bronchiolitis cases/100,000 (black) in southern Israel during 2004–05 through 2011–12 among (A) Bedouin children and (B) Jewish children. Bronchiolitis data were available through the end of the 2011 calendar year. Weekly influenza positive samples among Bedouin children (C) and Jewish children (D). RSV, respiratory syncytial virus.

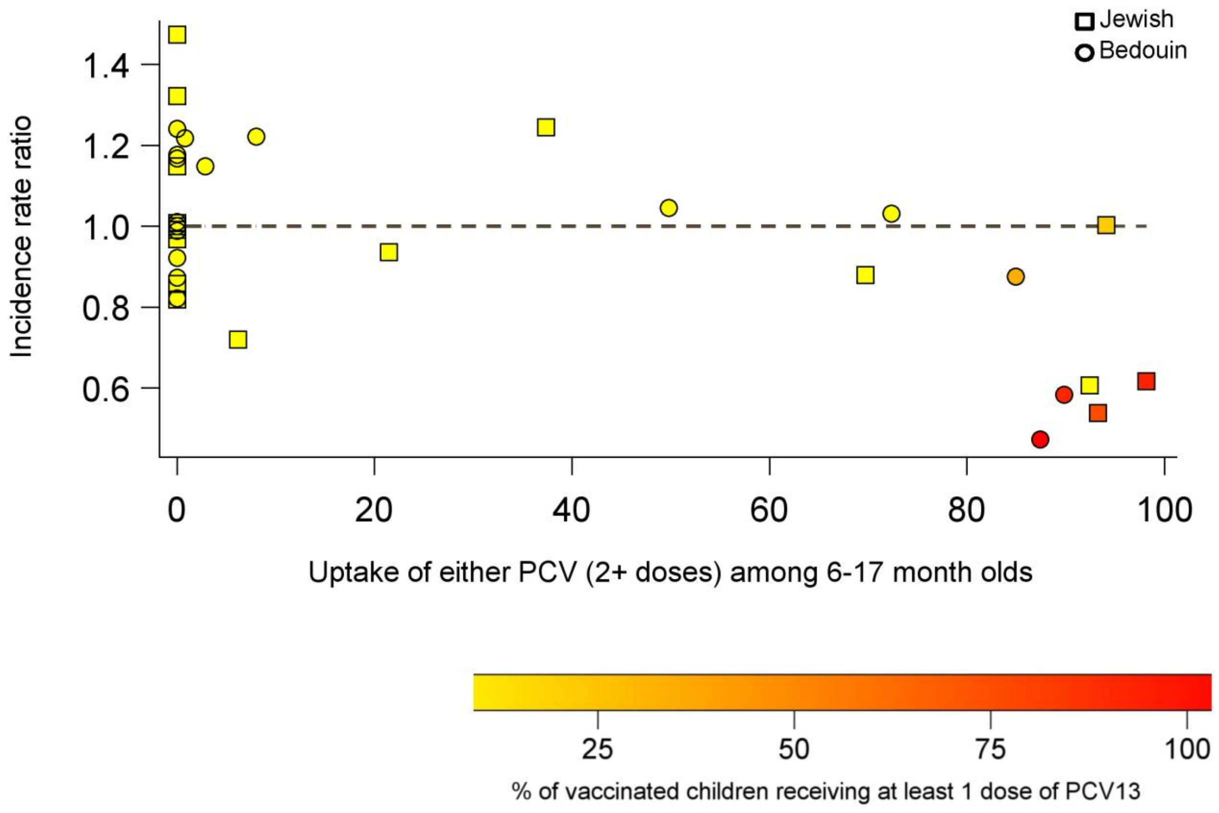

Technical Appendix Figure 4. Example of model fit and estimated contribution of influenza and RSV to RCAP incidence, southern Israel, among Bedouin children A) <6 months of age and B) 6–17 months of age. Grey line, observed RCAP incidence; red shading, estimated incidence attributed to RSV; blue shading, estimated increase attributed to influenza virus; gray shading, estimated incidence not attributed to RSV or influenza virus. RSV, respiratory syncytial virus; RCAP, radiologically confirmed alveolar pneumonia.
